# Primary mediastinal myelolipoma: A case report and review of the literature

**DOI:** 10.3892/ol.2012.1085

**Published:** 2012-12-19

**Authors:** CHUANYING GENG, NIAN LIU, GUANGZHONG YANG, MAN QI, WENGMING CHEN

**Affiliations:** 1 Departments of Hematology, Beijing Chaoyang Hospital, Capital Medical University, Beijing 100020, P.R. China; 2 Pathology, Beijing Chaoyang Hospital, Capital Medical University, Beijing 100020, P.R. China

**Keywords:** mediastinal, myelolipoma, diagnosis, treatment

## Abstract

Myelolipoma is a rare, benign neoplasm composed of mature adipocytes and hematopoietic tissue, mainly occurring in the adrenal glands. The majority of extra-adrenal myelolipomas have been identified in the presacral region and primary mediastinal myelolipoma is very rare. Computed tomography (CT) and magnetic resonance imaging (MRI) are effective methods to detect myelolipoma, while fine-needle aspiration (FNA) combined pathology is able to definitively rule out malignancy. There is no standard method of treatment for the disease. Small (<4 cm) asymptomatic tumors should be monitored, while symptomatic tumors or large (>7 cm) myelolipomas should be removed by surgery. This study describes a patient who presented with two mediastinal myelolipomas that were not encapsulated and presented as a string-of-pearls-type. The pathological diagnosis was myelolipoma and the patient did not relapse within the three years following resection.

## Introduction

Myelolipoma is a rare benign tumor composed of bone marrow elements admixed in an adipose tissue neoplasm, commonly originating from the adrenal gland (1). This type of tumor is typically discovered incidentally in the adrenal gland and occasionally in other regions, by pathologists (2). One of the rare anatomical sites of this tumor is the mediastinum. Approximately half of the extra-adrenal tumors originate from the presacral space, with the remainder arising from the perirenal, mediastinal, hepatic and gastric regions in decreasing order of frequency (3). We report a case of non-encapsulated mediastinal myelolipoma which was presented as a string-of-pearls-type, and diagnosed by pathological analysis. The study was approved by the Ethics Committee of Beijing Chaoyang Hospital, Capital Medical University, Beijing, China. Informed consent was obtained from the patient.

## Case report

A 68-year-old female with a history of anemia and well-controlled type 2 diabetes was admitted to the Beijing Chaoyang Hospital due to the presence of dull back pain and a cough for 8 months. There were no other symptoms or physical signs revealed by physical examination. The blood test results were as follows: Red blood cells, 2.57×10^12^/l; hemoglobin, 80 g/l; hematocrit, 23.80%; white blood cells, 5.67×10^9^/l and platelets, 206×10^9^/l. A bone marrow aspiration and biopsy demonstrated normal results.

**Figure 1:**
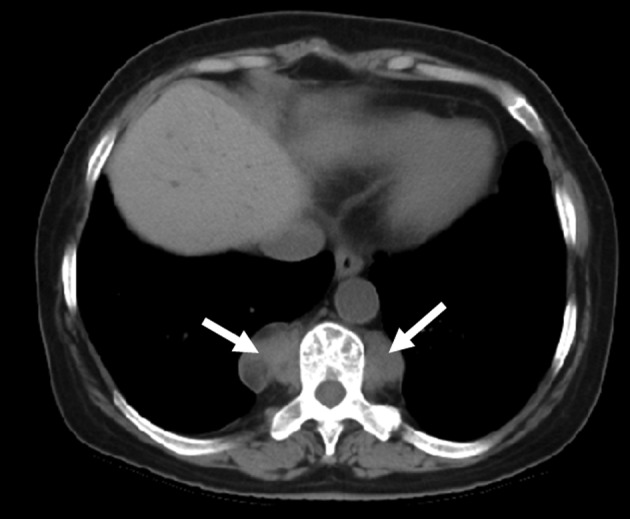
Chest computed tomography (CT) scan reveals two well-defined posterior mediastinal masses containing patchy areas of necrosis (arrows).

**Figure 2:**
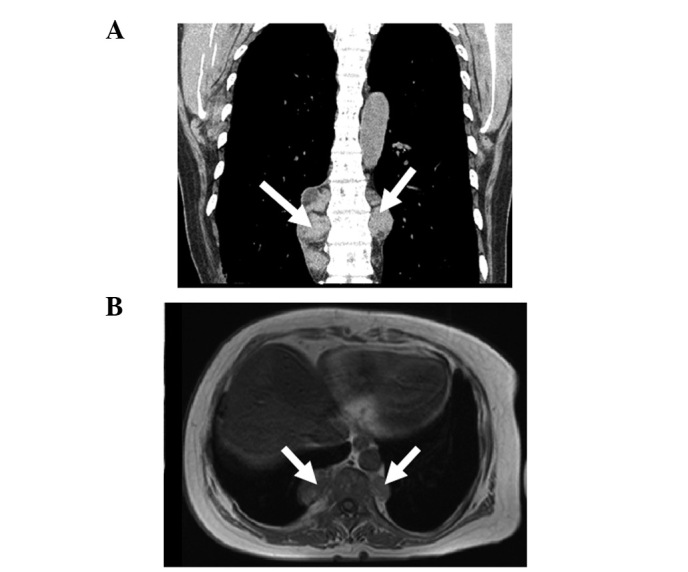
Magnetic resonance imaging (MRI) scan reveals a mass extending from levels T9-11 in the coronal planes. (A) The coronal plane of the MRI scan reveals two masses presenting beads (arrows). (B) The transverse plane of the MRI scan reveals two mediastinal masses (arrows).

A chest computed tomography (CT) scan revealed two posterior mediastinal masses. The masses were lobulated at the paravertebral region between the inferior lobes of the lung. The right posterior mediastinal mass measured ∼3.1×10 cm and the left was ∼2.5×9 cm. No evidence of bony erosion, pleural effusion or surrounding tissue infiltration was observed (Fig. 1). Magnetic resonance imaging (MRI) demonstrated a mass extending from levels T9-11 in the coronal planes. Mixed-signal lesions, mainly equal to a T1/T2 signal, were observed. In the marginal regions, a cystic lesion exhibited longer T1 and T2. There was no involvement of the vertebral canal and the bony structure was normal (Fig. 2). A CT scan revealed no abnormalities in the renal and adrenal glands.

**Figure 3:**
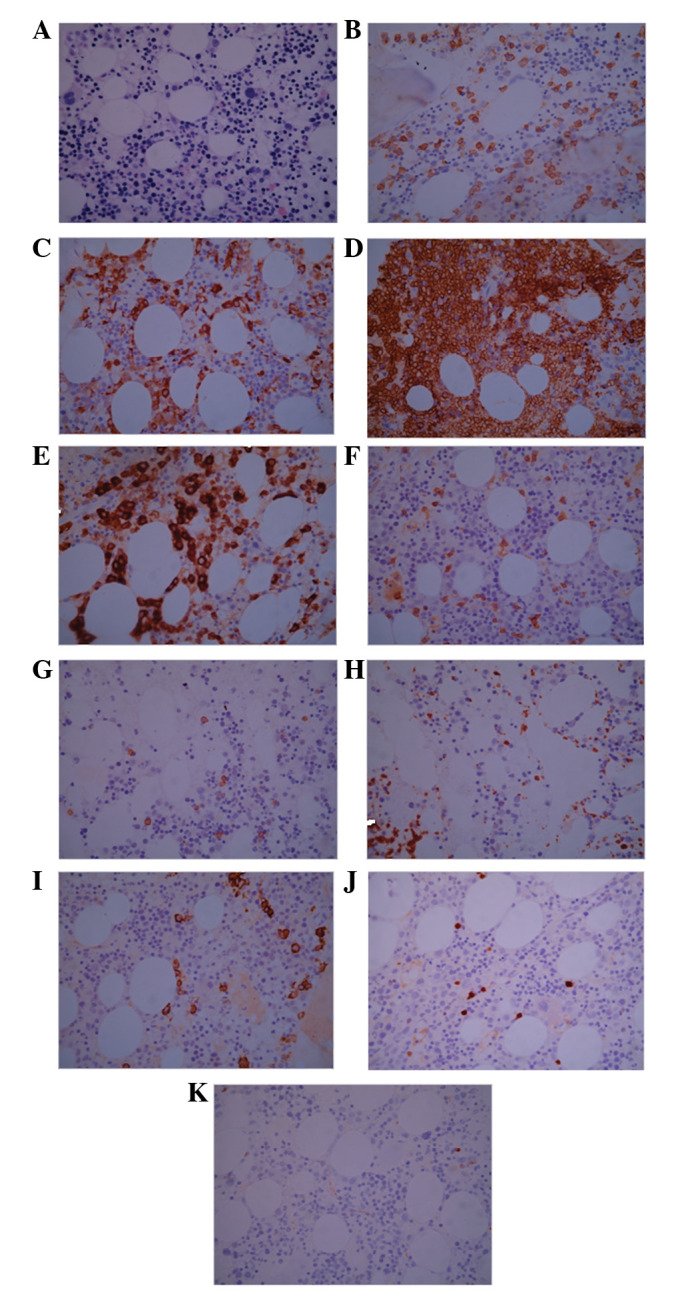
Hematoxylin and eosin (H&E) and immunohistochemical staining of the tumor tissue. (A) H&E staining reveals hematopoietic elements admixed with adipose tissue. Immunohistochemistry reveals CD15 (B); CD68 (C); CD235a (D); MPO (E); CD3 (F); CD20 (G); CD61 (H); CD138 (I); TdT (J) and CD34 (K) expression.

A standard left thoracotomy was performed and the intraoperative findings included string-of-pearls-type manifestation. The mass resembled fish flesh; it was not completely encapsulated and had clear boundaries. Tumor invasion of the vertebra was not observed. The pathological analysis indicated that the tumor was benign and a complete excision of the tumor was achieved. Pathological results revealed predominant mature adipose tissue with hematopoietic tissue. Microscopical analysis also demonstrated that the tumor comprised adipose tissue, together with hematopoietic tissue; features typical of myelolipoma (Fig. 3A). The pathological findings were consistent with a diagnosis of mediastinal extra-adrenal myelolipoma, and no signs of malignancy were observed. The immunohistochemistry results demonstrated that partial cells expressed CD15, CD235a, CD68 and MPO; minute cells expressed CD3, CD20, CD61 and CD138; district minority cells expressed TdT and no cells expressed CD34 (Fig. 3B-K). The patient was followed up over a 3-year period post-surgery, and has had no relapses at present.

## Discussion

Myelolipoma is a rare, benign neoplasm composed of mature adipocytes and hematopoietic tissue (4–7). The disease was first described in 1905, and was given the name ‘myelolipoma’ in 1929 (8). Myelolipomas mainly occur in the adrenal glands, where they are typically non-functioning and asymptomatic. The occurrence of the majority of extra-adrenal myelolipomas has been noted to be in the presacral region, while primary mediastinal myelolipoma is rare.

Myelolipomas are mesenchymal tumors composed of a mixture of adipose and hemopoietic tissue. Usually, myelolipomas are unilateral and asymptomatic. Myelolipomas are often <4 cm in diameter; however, larger lesions are capable of causing symptoms such as a mass effect or a hemorrhage (9). Symptoms associated with large myelolipomas are typically vague and include back or abdominal pain. At present, ultrasonography, CT and MRI are useful diagnostic tools and the incidental detection of myelolipoma has become more common (10). Although CT and MRI are effective in diagnosing myelolipoma, it is difficult to make a confident conclusion. Fine-needle aspiration (FNA) combined pathology is able to definitively rule out malignancy.

At present, there is no standard method of treatment for this disease. Daneshmand *et al*(10) suggested that small asymptomatic tumors (<4 cm in size) should be monitored, while symptomatic tumors or large myelolipomas (>7 cm in size) should be removed. The majority of myelolipomas are incidentally diagnosed by imaging detection and are not malignant. Therefore, treatment is not required and patients only need to be monitored regularly.

When myelolipomas grow in particular sites where they affect the function of important organs or induce certain symptoms that patients are not able to endure, surgical treatment is required to remove the myelolipomas. Myelolipomas are frequently resected by thoracoabdominal incision and may also be removed by laparoscopic surgery in certain experienced centers (11). Surgical excision is a useful method for treating myelolipomas, and the tumors generally do not recur.

In the present case, the patient was an elderly female who presented with dull back pain and a cough that had existed for 8 months. CT and MRI scans revealed two posterior mediastinal masses which presented as a string-of-pearls-type. However, no characteristics of extra-adrenal myelolipoma were pre-operatively observed. The patient exhibited symptoms and so the tumors were removed by thoracic surgery. Surgical resection followed by pathological analysis is an effective method for diagnosing rare tumors. The presence of megakaryocytes is essential for the diagnosis of myeloma (12). Pathological and immunohistochemical examination demonstrated that the mass of this patient was myelolipoma. In addition, the patient suffered from chronic moderate anemia for a further 20 years and the anemia did not improve following removal of the myelolipoma. The cause of the anemia was not clear; it may have been induced by immune factors.

We described a patient with two mediastinal myelolipomas that had been diagnosed by pathological analysis. Although the myelolipomas were not particularly large in diameter, they were both long and they stretched along the spine. Surgical treatment was effective, and the patient had no relapses for three years. Although primary mediastinal myelolipoma is rare, understanding its diagnosis and treatment are important. If a patient is suspected to have primary mediastinal myelolipoma by CT or MRI scans, this may be clarified it by pathological analysis. Subsequently, the tumor may be modified or removed by surgery, according to its effects on the body.

In conclusion, the present case demonstrates that CT and MRI scans are able to indicate the presence of mediastinal myelolipoma. Pathological analysis is an effective method to clarify the diagnosis. Observation and surgery are two regular treatment methods. Small, asymptomatic tumors should be monitored, while large tumors that cause unendurable symptoms may be removed by surgery.

## References

[1] Kenney PJ, Wagner BJ, Rao P, Heffess CS (1998). Myelolipoma: CT and pathologic features. Radiology.

[2] Kim K, Koo BC, Davis JT, Franco-Saenz R (1984). Primary myelolipoma of mediastinum. J Comput Tomogr.

[3] Kawanami S, Watanabe H, Aoki T, Nakata H, Hayashi T, Kido M (2000). Mediastinal myelolipoma: CT and MRI appearances. Eur Radiol.

[4] Dan D, Bahadursingh S, Hariharan S, Ramjit C, Naraynsingh V, Maharaj R (2012). Extra-adrenal perirenal myelolipoma. A case report and review of literature. G Chir.

[5] Akamatsu H, Koseki M, Nakaba H, Sunada S, Ito A, Teramoto S, Miyata M (2004). Giant adrenal myelolipoma: report of a case. Surg Today.

[6] Heylen S, Hubens G, Vaneerdeweg W (2011). Giant adrenal myelolipoma: a case report. Acta Chir Belg.

[7] Hasan M, Siddiqui F, Al-Ajmi M (2008). FNA diagnosis of adrenal myelolipoma: a rare entity. Diagn Cytopathol.

[8] Cha JS, Shin YS, Kim MK, Kim HJ (2011). Myelolipomas of both adrenal glands. Korean J Urol.

[9] Lawler LP, Pickhardt PJ (2001). Giant adrenal myelolipoma presenting with spontaneous hemorrhage. CT, MR and pathology correlation. Ir Med J.

[10] Daneshmand S, Quek ML (2006). Adrenal myelolipoma: diagnosis and management. Urol J.

[11] Schaeffer EM, Kavoussi LR (2005). Adrenal myelolipoma. J Urol.

[12] Gao B, Sugimura H, Sugimura S, Hattori Y, Iriyama T, Kano H (2002). Mediastinal myelolipoma. Asian Cardiovasc Thorac Ann.

